# Mixed Convective Flow and Heat Transfer of a Dual Stratified Micropolar Fluid Induced by a Permeable Stretching/Shrinking Sheet

**DOI:** 10.3390/e21121162

**Published:** 2019-11-27

**Authors:** Najiyah Safwa Khashi’ie, Norihan Md Arifin, Roslinda Nazar, Ezad Hafidz Hafidzuddin, Nadihah Wahi, Ioan Pop

**Affiliations:** 1 Institute for Mathematical Research, Universiti Putra Malaysia, Selangor 43400, Malaysia; najiyah@utem.edu.my; 2 Fakulti Teknologi Kejuruteraan Mekanikal dan Pembuatan, Universiti Teknikal Malaysia Melaka, Melaka 76100, Malaysia; 3 Department of Mathematics, Faculty of Science, Universiti Putra Malaysia, Selangor 43400, Malaysia; nadihah@upm.edu.my; 4 School of Mathematical Sciences, Faculty of Science and Technology, Universiti Kebangsaan Malaysia, Selangor 43600, Malaysia; rmn@ukm.edu.my; 5 Centre of Foundation Studies for Agricultural Science, Universiti Putra Malaysia, Selangor 43400, Malaysia; ezadhafidz@upm.edu.my; 6 Department of Mathematics, Babeş-Bolyai University, Cluj-Napoca R-400084, Romania; popm.ioan@yahoo.co.uk

**Keywords:** mixed convection, magnetohydrodynamics, micropolar fluid, stretching/shrinking sheet, double stratification, dual solutions

## Abstract

The present study accentuates the magnetohydrodynamics (MHD) flow and heat transfer characteristics of a dual stratified micropolar fluid over a permeable stretching/shrinking sheet. Thermal and solutal buoyancy forces are also included to incorporate with the stratification effect. Similarity, transformation is applied to reduce the governing model (partial differential equations) into a set of nonlinear ordinary differential equations (ODEs) due to its complexity. Using bvp4c solver in the MATLAB software, numerical results for some limiting cases are in favorable agreement with the earlier published results. Both assisting and opposing buoyancy flows have dual similarity solutions within specific range of suction and stretching/shrinking parameters, whereas only a distinctive solution is observed for pure forced convective flow. The micropolar fluid shows a disparate pattern of flow, heat and mass transfer characteristics between stretching and shrinking cases. Unlike the shrinking flow, the surface velocity gradient, local Nusselt and Sherwood numbers for stretching flow intensify with the increment of the material parameter. The result from stability analysis reveals that the first solution is the real solution, whereas the second solution is virtual.

## 1. Introduction

Nowadays, energy generation becomes a major issue in industrial requirements. Many attentions are focused to increase the heat transfer rate of the systems which include power stations, chemical plants, air conditioning and petrochemical industry. Suction is one of the boundary layer control methods, which is traditionally used in drag reduction of bodies in an external flow or energy losses in channels [1,2,3,4]. The imposition of suction on the boundary layer flow due to a shrinking sheet was initially explored by Miklavčič and Wang [5] for a viscous fluid. Unlike the stretching flow, the flow towards a shrinking sheet (shrinking flow) is distracted away from the sheet due to the unconfined vorticity. Hence, an application of suitable wall mass suction can effectively be used to stabilize the vorticity within the boundary layer and, subsequently, delay the boundary layer separation. In addition, Miklavčič and Wang [5] proved that the similarity dual solutions were attainable for higher magnitude of the suction parameter S>2 while zero solution for S<2. However, the result was only conclusive to the viscous fluid with the absence of any physical parameters.

Many engineering processes deal with non-Newtonian fluids, which cannot be described by the traditional viscous fluid model. In general, non-Newtonian fluids can be categorized into dilatant (shear thickening) fluid and pseudoplastic (shear thinning) fluid. Nonetheless, the micropolar fluid as theoretically described by Eringen [6] is a fluid with microstructures and belongs to the nonsymmetric stress tensor. In other words, micropolar fluid model exhibits the microscopic effects due to the micromotion and the local structure of the fluid elements. Few examples of liquids which can be represented by micropolar fluid model are liquid crystals, animal blood, polymer suspension and lubricants. The application of micropolar fluid in blood flow model were conducted by Ellahi et al. [7], Mekheimer et al. [8] and Mekheimer and El Kot [9], while Bitla and Iyengar [10] investigated the pulsating flow between two homogeneous permeable beds using micropolar fluid. The study by Bitla and Iyengar [10] might provide a deep insight of porous stretching sheet in micropolar theory. The analysis of micropolar fluid flow over a shrinking sheet was conducted by Yacob and Ishak [11]. The results show that the paired solutions were observed for both viscous and micropolar fluids within a specific range of the suction parameter. In addition, stronger suction was needed to generate the solutions for a micropolar fluid as compared to the viscous fluid. Rosali et al. [12] studied the stagnation point flow of micropolar fluid past a permeable stretching/shrinking sheet saturated in a porous medium. Since the free stream velocity was considered, small value of suction $\left(0.5\leq S\leq 0.8\right)$ also could generate two similarity solutions within a certain range of the shrinking parameter. The MHD stagnation point flow of micropolar fluid over a stretching/shrinking surface with velocity slip was examined by Soid et al. [13]. The stability analysis was executed to test the reliability of the non-unique solutions and it was revealed that the first solution was a stable flow. Due to the technological advancement and the significance of the micropolar fluid in industrial applications, many further studies were conducted with the imposition of relevant physical parameter. The buoyancy induced flow due to the variations of wall surface temperature also has been a major area of interest for most researchers. Lund et al. [14] obtained dual solutions for MHD mixed convective flow, heat and mass transfer of micropolar nanofluid over a shrinking surface. The dual solutions were attainable for assisting flow case when stronger suction (S>2) was used for each values of micropolar parameter. Further, the MHD mixed convective flow and heat transfer of micropolar nanofluid over a static wedge was scrutinized by Zaib and Haq [15] and they also possessed two solutions. Recent investigations on the boundary layer flow of micropolar fluid with buoyancy effect were analyzed by Sandeep and Sulochana [16], Rashidi et al. [17], Zaib et al. [18], Waqas et al. [19], Turkyilmazoglu [20], Patel and Singh [21] and Jusoh et al. [22].

Fluid stratification phenomena are defined as the variation in fluid temperature, concentration or the appearance of contrasting fluids. There are many engineering applications involving thermal stratification; e.g., the one reported by Bouhal et al. [23] and Liu et al. [24]. In addition, the analysis of double stratification (temperature and concentration) has also been subject of interests for many researchers. There is also a connection between stratification and convection processes under this phenomenon [25]. Khashi’ie et al. [26] investigated the dual and stable solutions of MHD mixed convective stagnation point flow with double stratification impact using viscous fluid model. The thermal and solutal stratification parameter reduced the liquid temperature and concentration, accordingly, while both temperature and concentration profiles diminished with the augmentation of the magnetic and buoyancy parameters. Khashi’ie et al. [27] emphasized the MHD flow and heat transfer with dual stratification effects over a permeable shrinking/stretching sheet using Buongiorno’s model of nanofluid. The appearance of dual solutions were noticed with the imposition of suction parameter. Unlike Khashi’ie et al. [26], the nanoliquid temperature and concentration profiles slightly inclined with the increment of thermal and solutal stratification parameters, respectively. This might be due to the application of strong suction and nanoparticles. Only a few works reported the boundary layer flow of a micropolar fluid in a stratified medium. Srinivasacharya and Upendar [28] considered the mutual effects of magnetic field, thermal and solutal stratifications on the free convective micropolar fluid flow. The results show that an increment in the magnetic parameter might lessen the velocity, heat and mass transfer rates. Mishra et al. [29] extended the work of Srinivasacharya and Upendar [28] by including the heat source effect. Rashad et al. [30] found that both skin friction coefficient and Nusselt number increased with a boost in thermal stratification, however a contrary result was obtained for the Sherwood number when the solutal stratification was enhanced. Recently, Koriko et al. [31] studied the MHD micropolar fluid flow towards a vertical permeable stretching sheet with the presence of double stratification and nonlinear thermal radiation. The analysis showed that both fluid velocity and temperature decreased with an upsurge of thermal stratification parameter. The influence of nonlinear thermal radiation, second-order slip and magnetic field on the dual stratified micropolar fluid flow past a vertical stretching sheet was investigated by Sarojamma et al. [32]. They revealed that there was an undershoot in the fluid temperature (negative temperature) due to a strong magnitude of thermal stratification parameter. In addition, an excessive of mass stratification parameter and higher value of Prandtl number (weaker thermal diffusivity) also showed an undershoot in the fluid concentration.

Inspired by the previous literature, the present work is mainly focused on the MHD flow, heat and mass transfer of micropolar fluid induced by a vertical permeable shrinking/stretching sheet in a dual stratified medium. The previously published results show that dual (non-unique) solutions are feasible for the case of assisting and opposing flows induced by a static, stretching or shrinking surface. Hence, the duality and stability of solutions are also the main objectives of the present work. The authors are confident that there are no published works which discuss the present results. In addition, there are many works that discuss the fixed (constant) wall and ambient temperatures/concentrations but, in the real industrial and engineering processes, both wall and ambient temperatures/concentrations can be in variable form (stratification). Hence, the authors believe that the present results are significant to other researchers with different backgrounds (mathematics/engineering/physics).

## 2. Mathematical Formulation

**Figure 1:**
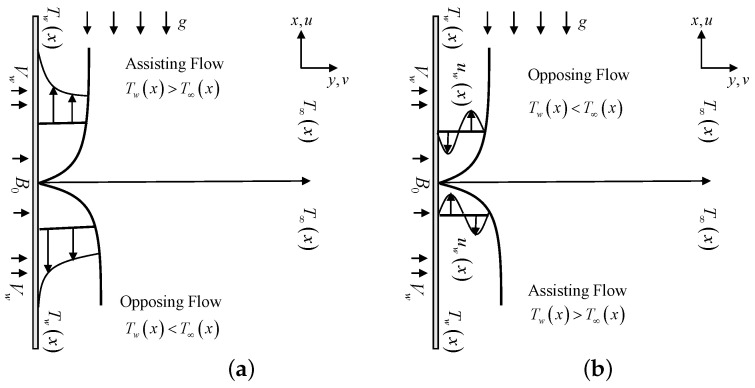
Physical model and coordinate system of: (**a**) stretching case; and (**b**) shrinking case.

Consider a steady and laminar flow of an incompressible micropolar fluid past a shrinking/stretching sheet with a linear velocity, $u_{w}\left(x\right)=ax$, as depicted in Figure 1. The sheet is also permeable for possible wall mass fluid suction/injection with variable wall temperature $T_{w}\left(x\right)=T_{0}+Ax$ and $C_{w}\left(x\right)=C_{0}+Ex$ such that $T_{w}>T_{0}$ and $C_{w}>C_{0}$. Further, $T_{\infty }\left(x\right)=T_{0}+Bx$ and $C_{\infty }\left(x\right)=C_{0}+Fx$ are in a linear stratified form where *B* and *F* are constants [28].

An applied magnetic field with constant strength is perpendicular to the flow direction, whereas the magnetic Reynolds number is sufficiently small and, consequently, the induced magnetic field is ignored. Under the boundary layer approximations and all the physical model assumptions, the simplified PDEs are (see Rashad et al. [30], Sarojamma et al. [32]):

(1) $$\frac{\partial u}{\partial x}+\frac{\partial v}{\partial y}=0,$$

(2) $$\rho \left(u\frac{\partial u}{\partial x}+v\frac{\partial u}{\partial y}\right)=\left(\mu +\kappa \right)\frac{\partial^{2}u}{\partial y^{2}}+\kappa \frac{\partial \omega }{\partial y}-\sigma_{M}{B_{0}}^{2}u+\rho g\left[\beta_{T}\left(T-T_{\infty }\right)+\beta_{c}\left(C-C_{\infty }\right)\right],$$

(3) $$\rho j\left(u\frac{\partial \omega }{\partial x}+v\frac{\partial \omega }{\partial y}\right)=\gamma \frac{\partial^{2}\omega }{\partial y^{2}}-\kappa \left(2\omega +\frac{\partial u}{\partial y}\right),$$

(4) $$u\frac{\partial T}{\partial x}+v\frac{\partial T}{\partial y}=\alpha \frac{\partial^{2}T}{\partial y^{2}},$$

(5) $$u\frac{\partial C}{\partial x}+v\frac{\partial C}{\partial y}=D\frac{\partial^{2}C}{\partial y^{2}},$$

where *u* and *v* are the velocities along *x*- and *y*-directions, respectively; *T* is the fluid temperature; *C* is the fluid concentration; and *g* is the gravitational acceleration. Further, $\beta_{T}$ and $\beta_{C}$ are the coefficient of thermal and solutal expansions, μ and ν are the fluid dynamic and kinematic viscosities, and α and *D* are the thermal and molecular diffusivities, respectively. The initial and boundary conditions are:

(6) u(x,0)=εuwx,v(x,0)=Vw,ω(x,0)=−m∂u∂u∂y∂y,T(x,0)=Tw(x),C(x,0)=Cw(x),

(7) $$u\left(x,\infty \right)\to 0,\;\omega \left(x,\infty \right)\to 0,\;T\left(x,\infty \right)\to T_{\infty }\left(x\right),\;C\left(x,\infty \right)\to C_{\infty }\left(x\right),$$

In the initial condition in Equation (6), *m* is a constant where 0≤m≤1;m=0 is referred as strong concentration by Guram and Smith [33]. The case *m* = 1/2 denotes the weak concentration [34], whereas m=1, as suggested by Peddieson [35], is used for the turbulent boundary layer model. Furthermore, $V_{w}$ is the mass flux through the sheet where $V_{w}=-\sqrt{a\nu }S$ and

(8) $$V_{w}=\left\{\begin{array}{c} V_{w}<0\;;\;\mathrm{suction}\\ V_{w}>0\;;\;\mathrm{injection} \end{array}\right.$$

The governing PDEs in Equations (1)–(5) subject to the boundary conditions in Equations (6) and (7) can be simplified into a system of ODEs using the following similarity transformations where η and ψ are the similarity variable and the stream function, respectively.

(9) $$\eta =\sqrt{\frac{a}{\nu }}y,\;\psi =\sqrt{a\nu }xf\left(\eta \right),\;\omega =ax\sqrt{\frac{a}{\nu }}g\left(\eta \right),\;\theta \left(\eta \right)=\frac{T-T_{\infty }\left(x\right)}{T_{w}\left(x\right)-T_{0}},\;\phi \left(\eta \right)=\frac{C-C_{\infty }\left(x\right)}{C_{w}\left(x\right)-C_{0}}.$$

Further, u=∂ψ∂ψ∂y∂y and v=−∂ψ∂ψ∂x∂y, which complies with Equation (1). Hence, the transformed ODEs are:

(10) $$f^{\prime \prime \prime }=\frac{1}{1+K}\left[{\left(f^{\prime }\right)}^{2}-ff^{\prime \prime }-Kg^{\prime }+Mf^{\prime }-\lambda \theta -N\phi \right],$$

(11) $$g^{\prime \prime }=\frac{1}{\left(1+\frac{K}{2}\right)}\left[f^{\prime }g-fg^{\prime }+K\left(2g+f^{\prime \prime }\right)\right],$$

(12) $$\theta^{\prime \prime }=-\mathrm{Pr}\left[f\theta^{\prime }-\left(\theta +\delta_{1}\right)f^{\prime }\right],$$

(13) $$\phi^{\prime \prime }=-Sc\left[f\phi^{\prime }-\left(\phi +\delta_{2}\right)f^{\prime }\right],$$

where K=κκμμ; K=0 and K>0 correspond to the Newtonian and micropolar fluid, respectively; Gr=gβTΔTx3gβTΔTx3ν2ν2 is the local Grashof number due to temperature; Gr*=gβCΔCx3gβCΔCx3ν2ν2 is the local Grashof number due to concentration; Rex=xuw(x)xuw(x)νν is the local Reynolds number; λ=GrGrRex2Rex2 and N=Gr*Gr*Rex2Rex2 are the thermal and solutal buoyancy parameters, respectively; M=σMB02σMB02ρaρa is the magnetic parameter; Sc=ννDD is the Schmidt number; and δ1=BAA and δ2=FEE are the thermal and solutal stratification parameters, respectively; In addition, λ>0 refers to the assisting flow, λ<0 indicates the opposing flow and λ=0 is the pure forced convective flow. The simplified initial and boundary conditions are

(14) $$f\left(0\right)=S,\;f^{\prime }\left(0\right)=\varepsilon ,\;g\left(0\right)=-mf^{\prime \prime }\left(0\right),\;\theta \left(0\right)=1-\delta_{1},\;\phi \left(0\right)=1-\delta_{2},$$

(15) $$f^{\prime }\left(\infty \right)\to 0,\;g\left(\infty \right)\to 0,\;\theta \left(\infty \right)\to 0,\;\phi \left(\infty \right)\to 0,$$

respectively. The physical quantities that are used in the study are defined as

(16) $$C_{f}=\frac{\tau_{w}}{\rho u_{w}^{2}},\;M_{w}=\frac{m_{w}}{\rho xu_{w}^{2}},\;Nu_{x}=\frac{xq_{w}}{k(T_{w}\left(x\right)-T_{0})},\;Sh_{x}=\frac{xq_{m}}{D(C_{w}\left(x\right)-C_{0})},$$

respectively, where $\tau_{w}$ is the wall shear stress along the stretching/shrinking surface, $m_{w}$ is the wall couple stress, *k* is the thermal conductivity of the fluid, $q_{w}$ is the surface heat flux and $q_{m}$ is the surface mass flux.

(17) $$\tau_{w}={\left[\left(\mu +\kappa \right)\left(\frac{\partial u}{\partial y}\right)+\kappa \omega \right]}_{y=0},\;m_{w}=\gamma {\left(\frac{\partial \omega }{\partial y}\right)}_{y=0},\;q_{w}=-k{\left(\frac{\partial T}{\partial y}\right)}_{y=0},\;q_{m}=-D{\left(\frac{\partial C}{\partial y}\right)}_{y=0}.$$

The reduced skin friction coefficient $R{e_{x}}^{1/2}C_{f}$, local Nusselt $R{e_{x}}^{-1/2}Nu_{x}$ and Sherwood $R{e_{x}}^{-1/2}Sh_{x}$ numbers in the dimensionless form are given by

(18) $$\begin{array}{c} R{e_{x}}^{1/2}C_{f}=\left(1+\left(1-m\right)K\right)f^{\prime \prime }\left(0\right),\;Re_{x}M_{w}=\left(1+\frac{K}{2}\right)g^{\prime }\left(0\right),\;\\ R{e_{x}}^{-1/2}Nu_{x}=-\theta^{\prime }\left(0\right),\;R{e_{x}}^{-1/2}Sh_{x}=-\phi^{\prime }\left(0\right). \end{array}$$

## 3. Stability Analysis

The stability analysis is important to test the reliability of the non-unique similarity solutions. In general, depending on the assumptions of physical model, a boundary layer problem may produce zero, unique or multiple solutions. For example, if the problem has non-unique solutions but the researchers manage to find one solution only, there is a probability that the solution is the lower branch solution (unstable/not real). This will lead to the misinterpretation of the flow and heat transfer characteristics. Depending on the numerical methods used (shooting/Keller-box/bvp4c function in MATLAB), the dual solutions in the present work are searched by assuming two sets of initial guesses (one set for first solution and another for second solution), which will produce two disparate profiles (velocity/temperature/concentration). Both the first and second profiles must asymptotically fulfill the far field boundary conditions (see Equation (15)). The first (upper branch) solution is denoted for the first solution which satisfies the far field boundary condition. The current studies that discussed the stability formulation and analysis were reported by Khashi’ie et al. [26,27], Bakar et al. [36,37], Yahaya et al. [38], Salleh et al. [39,40] and Jamaluddin et al. [41]. Additionally, the stability formulation for micropolar fluid flow towards a stretching/shrinking surface was recently reported by Soid et al. [13] and Lok et al. [42].

Physically, the solution is virtual or not real if there exist a growth of disturbance in the solution. Initiated by Merkin [43], the first stage in the stability analysis is to consider an unsteady (flow that change with time) problem since the disturbance may decay or grow with time. Hence, an unsteady form of Equations (2)–(5) are

(19) $$\frac{\partial u}{\partial t}+u\frac{\partial u}{\partial x}+v\frac{\partial u}{\partial y}=\left(\frac{\mu +\kappa }{\rho }\right)\frac{\partial^{2}u}{\partial y^{2}}+\frac{\kappa }{\rho }\frac{\partial \omega }{\partial y}-\frac{\sigma_{M}{B_{0}}^{2}}{\rho }u+g\left[\beta_{T}\left(T-T_{\infty }\right)+\beta_{c}\left(C-C_{\infty }\right)\right],$$

(20) $$\frac{\partial \omega }{\partial t}+u\frac{\partial \omega }{\partial x}+v\frac{\partial \omega }{\partial y}=\frac{\gamma }{\rho j}\frac{\partial^{2}\omega }{\partial y^{2}}-\frac{\kappa }{\rho j}\left(2\omega +\frac{\partial u}{\partial y}\right),$$

(21) $$\frac{\partial T}{\partial t}+u\frac{\partial T}{\partial x}+v\frac{\partial T}{\partial y}=\alpha \frac{\partial^{2}T}{\partial y^{2}},$$

(22) $$\frac{\partial C}{\partial t}+u\frac{\partial C}{\partial x}+v\frac{\partial C}{\partial y}=D\frac{\partial^{2}C}{\partial y^{2}}.$$

The new similarity transformation for the unsteady case is introduced where τ=at is the dimensionless time variable

(23) $$\begin{array}{c} \eta =\sqrt{\frac{a}{\nu }}y,\;\psi =\sqrt{a\nu }xf\left(\eta ,\tau \right),\;\omega =ax\sqrt{\frac{a}{\nu }}g\left(\eta ,\tau \right),\;\theta \left(\eta ,\tau \right)=\frac{T-T_{\infty }\left(x\right)}{T_{w}\left(x\right)-T_{0}},\\ \phi \left(\eta ,\tau \right)=\frac{C-C_{\infty }\left(x\right)}{C_{w}\left(x\right)-C_{0}},\;u=ax\frac{\partial f}{\partial \eta }\left(\eta ,\tau \right),\;v=-\sqrt{a\nu }f\left(\eta ,\tau \right). \end{array}$$

Using Equation (23), Equations (19)–(22) are transformed into

(24) $$\left(1+K\right)\frac{\partial^{3}f}{\partial \eta^{3}}+f\frac{\partial^{2}f}{\partial \eta^{2}}-{\left(\frac{\partial f}{\partial \eta }\right)}^{2}-\frac{\partial^{2}f}{\partial \eta \partial \tau }-M\frac{\partial f}{\partial \eta }+K\frac{\partial g}{\partial \eta }+\lambda \theta +N\phi =0,$$

(25) $$\left(1+\frac{K}{2}\right)\frac{\partial^{2}g}{\partial \eta^{2}}+f\frac{\partial g}{\partial \eta }-g\frac{\partial f}{\partial \eta }-\frac{\partial g}{\partial \tau }-K\left(2g+\frac{\partial^{2}f}{\partial \eta^{2}}\right)=0,$$

(26) $$\frac{1}{\mathrm{Pr}}\frac{\partial^{2}\theta }{\partial \eta^{2}}+f\frac{\partial \theta }{\partial \eta }-\left(\theta +\delta_{1}\right)\frac{\partial f}{\partial \eta }-\frac{\partial \theta }{\partial \tau }=0,$$

(27) $$\frac{1}{Sc}\frac{\partial^{2}\phi }{\partial \eta^{2}}+f\frac{\partial \phi }{\partial \eta }-\left(\phi +\delta_{2}\right)\frac{\partial f}{\partial \eta }-\frac{\partial \phi }{\partial \tau }=0,$$

with the conditions

(28) $$\begin{array}{c} f\left(0,\tau \right)=S,\;\frac{\partial f}{\partial \eta }\left(0,\tau \right)=\varepsilon ,\;g\left(0,\tau \right)=0,\;\theta \left(0,\tau \right)=1-\delta_{1},\;\phi \left(0,\tau \right)=1-\delta_{2},\\ \frac{\partial f}{\partial \eta }\left(\eta ,\tau \right)\to 0,\;g\left(\eta ,\tau \right)\to 0\theta \left(\eta ,\tau \right)\to 0,\;\phi \left(\eta ,\tau \right)\to 0\;\mathrm{as}\;\eta \to \infty \end{array}$$

The following representation (see Equation (29)) is used to determine the behavior of the solution’s stability by perturbing with the disturbance (see Weidman et al. [44]). For the stability process, steady flow solutions $f\left(\eta \right)=f_{0}\left(\eta \right)$, $g\left(\eta \right)=g_{0}\left(\eta \right)$, $h\left(\eta \right)=h_{0}\left(\eta \right)$, $k\left(\eta \right)=k_{0}\left(\eta \right)$, $\theta \left(\eta \right)=\theta_{0}\left(\eta \right)$ and $\phi \left(\eta \right)=\phi_{0}\left(\eta \right)$, which have satisfied the boundary value problem in Equations (10)–(17), are examined by the following expressions

(29) $$\left.\begin{array}{c} f\left(\eta ,\tau \right)=f_{0}\left(\eta \right)+e^{-\sigma \tau }F\left(\eta \right)\\ g\left(\eta ,\tau \right)=g_{0}\left(\eta \right)+e^{-\sigma \tau }G\left(\eta \right)\\ \theta \left(\eta ,\tau \right)=\theta_{0}\left(\eta \right)+e^{-\sigma \tau }P\left(\eta \right)\\ \phi \left(\eta ,\tau \right)=\phi_{0}\left(\eta \right)+e^{-\sigma \tau }R\left(\eta \right) \end{array}\right\},$$

where $f\left(\eta \right)=f_{0}\left(\eta \right)$, $g\left(\eta \right)=g_{0}\left(\eta \right)$, $\theta \left(\eta \right)=\theta_{0}\left(\eta \right)$ and $\phi \left(\eta \right)=\phi_{0}\left(\eta \right)$ are the steady state solutions; σ is an anonymous eigenvalue parameter; and F(η), G(η), P(η) and R(η) are small relative to the steady solutions $f_{0}\left(\eta \right)$, $g_{0}\left(\eta \right)$, $\theta_{0}\left(\eta \right)$ and $\phi_{0}\left(\eta \right)$, proportionately. The following linearized eigenvalue problem is attained by substituting Equation (29) into Equations (24)–(28):

(30) $$\left(1+K\right){F_{0}}^{\prime \prime \prime }+f_{0}{F_{0}}^{\prime \prime }+{f_{0}}^{\prime \prime }F_{0}-\left(2{f_{0}}^{\prime }-\sigma +M\right){F_{0}}^{\prime }+K{G_{0}}^{\prime }+\lambda P_{0}+NR_{0}=0,$$

(31) $$\left(1+\frac{K}{2}\right){G_{0}}^{\prime \prime }+f_{0}{G_{0}}^{\prime }+F_{0}{g_{0}}^{\prime }-g_{0}{F_{0}}^{\prime }-G_{0}{f_{0}}^{\prime }+\sigma G_{0}-K\left(2G_{0}+{F_{0}}^{\prime \prime }\right)=0,$$

(32) $$\frac{1}{\mathrm{Pr}}{P_{0}}^{\prime \prime }+F_{0}{\theta_{0}}^{\prime }+f_{0}{P_{0}}^{\prime }-\left(\theta_{0}+\delta_{1}\right){F_{0}}^{\prime }-P_{0}{f_{0}}^{\prime }+\sigma P_{0}=0,$$

(33) $$\frac{1}{Sc}{R_{0}}^{\prime \prime }+F_{0}{\phi_{0}}^{\prime }+f_{0}{R_{0}}^{\prime }-\left(\phi_{0}+\delta_{2}\right){F_{0}}^{\prime }-R_{0}{f_{0}}^{\prime }+\sigma R_{0}=0,$$

(34) $$\begin{array}{c} F_{0}\left(0\right)=0,\;{F_{0}}^{\prime }\left(0\right)=0,\;G_{0}\left(0\right)=0,\;P_{0}\left(0\right)=0,\;R_{0}\left(0\right)=0,\\ {F_{0}}^{\prime }\left(\eta \right)\to 0,\;G_{0}\left(\eta \right)\to 0,\;P_{0}\left(\eta \right)\to 0,\;R_{0}\left(\eta \right)\to 0\;\mathrm{as}\;\eta \to \infty \end{array}$$

In the bvp4c solver, the boundary condition $F^{\prime }\left(\eta \right)\to 0$ as η→∞ is renewed with the normalizing boundary condition $F^{\prime \prime }\left(0\right)=1$ to obtain the possible range of smallest eigenvalue, $\sigma_{1}$, as proposed by Harris et al. [45]. It is worth pointing out that a positive $\sigma_{1}$ implies that the solution is stable, whereas a negative value of $\sigma_{1}$ signifies that the disturbance occurs in the solution and, thus, the solution is irrational.

## 4. Results and Discussion

The similarity solutions were attained by solving Equations (10)–(13) with the transformed conditions in Equations (14) and (15) using the bvp4c code in the MATLAB software. The numerical procedure of bvp4c code for solving steady flow problem and stability analysis was elaborated by Yahaya et al. [37]. The values of m=0, Pr=7, Sc=0.78 and N=1 were fixed in the entire computation, whereas the boundary layer thickness for both first and second solutions was $\eta_{\infty }=15$. Table 1 elucidates the comparison of few values $\left(-\theta^{\prime }\left(0\right)\;\mathrm{and}\;-\phi^{\prime }\left(0\right)\right)$ with Srinivasacharya and Upendar [28] when K=Pr=λ=N=1, $\delta_{2}=Sc=0.2$, S=ε=0, $\delta_{1}=0.1$ and η=25. Unlike the other works on boundary layer of micropolar fluid flow (see [11,12,13,14,15,16,17,18,19,20,21,22,30,31,32]), Srinivasacharya and Upendar [28] used a model with coupling number to represent the micropolar fluid. However, using the limiting values of the physical parameters, we manage to compare the present results with those by Srinivasacharya and Upendar [28] as presented in Table 1. The present and previous results are in a positive agreement and the approximate percent relative error $\varepsilon_{a}$ are also small and acceptable (<0.5%).

**Figure 2:**
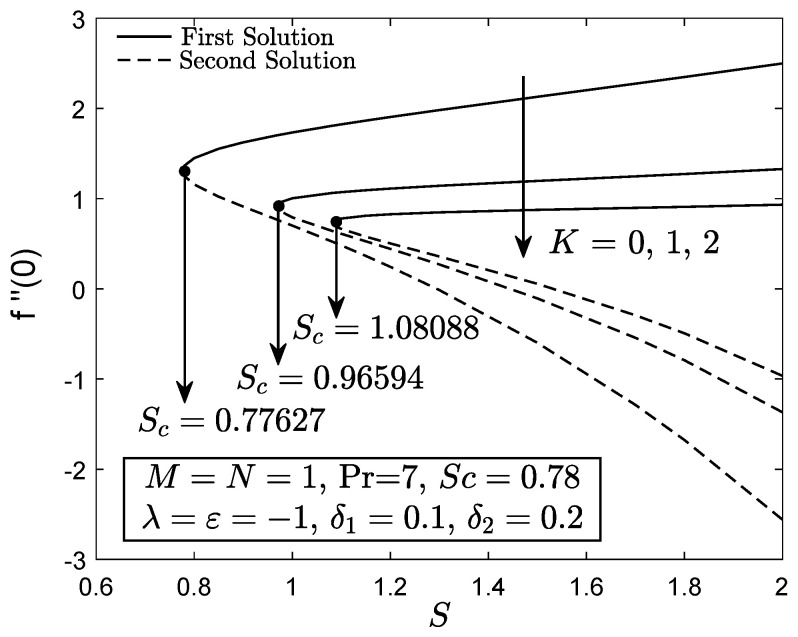
$f^{\prime \prime }\left(0\right)$ towards *S* for disparate values of *K*.

**Figure 3:**
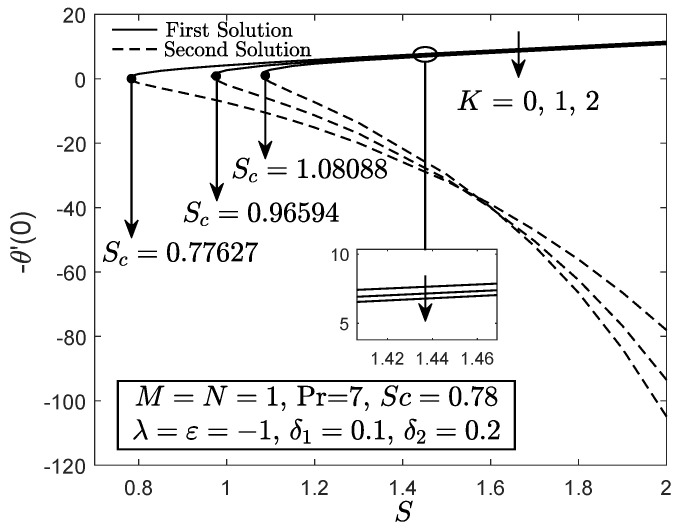
$-\theta^{\prime }\left(0\right)$ towards *S* for disparate values of *K*.

**Figure 4:**
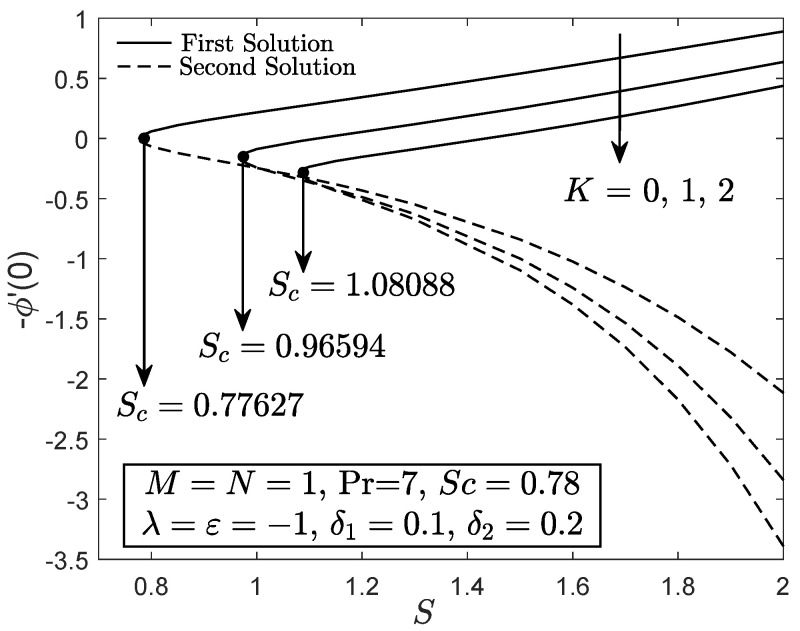
$-\phi^{\prime }\left(0\right)$ towards *S* for disparate values of *K*.

Figure 2, Figure 3 and Figure 4 exhibit the variation of $f^{\prime \prime }\left(0\right)$, $-\theta^{\prime }\left(0\right)$ and $-\phi^{\prime }\left(0\right)$ with *S*, respectively. These figures reveal the minimum value of the suction parameter *S* that is decisive to obtain dual solutions for both shrinking (ε=−1) and opposing (λ=−1) flow case of both viscous (K=0) and micropolar (K>0) fluids. The critical value $S_{c}$ is known as the joining point of the upper branch (physical) and lower branch solutions, whereas no solution is feasible for $S<S_{c}$. In addition, higher value of suction is required for a micropolar fluid to exist in both shrinking and opposing flow case under dual stratified medium as compared to the viscous fluid. However, unlike the critical value of suction obtained by Yacob and Ishak [11] for all material parameter, the minimum suction value in the present work is small (S<2). It seems that the mixed convection and stratification processes has significant effect in reducing the excessive amount of suction. However, the results may be different if the aiding flow (λ>0) case is considered since the enhancement of the buoyancy parameter can accelerate the fluid movement. Further, it is also apparent that $f^{\prime \prime }\left(0\right)$, $-\theta^{\prime }\left(0\right)$ and $-\phi^{\prime }\left(0\right)$ as portrayed in Figure 2, Figure 3 and Figure 4 reduce with the addition of the material parameter *K* for each value of *S*. The characteristics of surface velocity gradient $f^{\prime \prime }\left(0\right)$ and temperature gradient $-\theta^{\prime }\left(0\right)$ as *K* increases for each value of *S* are in accordance with those by Yacob and Ishak [11]. As the material/micropolar parameter enhances, the fluid viscosity weakens and subsequently, lessens the surface velocity gradient $f^{\prime \prime }\left(0\right)$ [32]. However, the results in Figure 2, Figure 3 and Figure 4 are only applicable for the flow due to the shrinking sheet. This makes the micropolar fluid useful in drag reduction of the shrinking flow.

**Figure 5:**
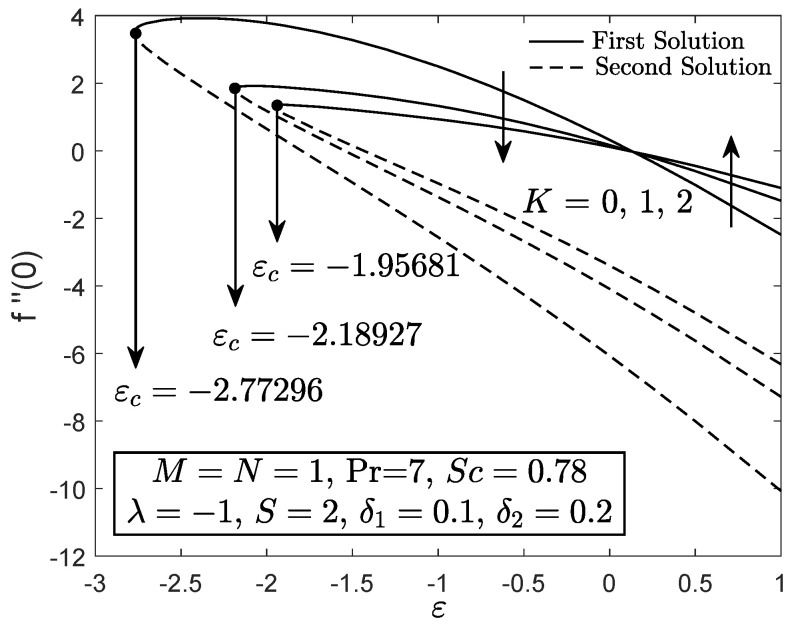
$f^{\prime \prime }\left(0\right)$ towards ε for viscous fluid (K=0) and micropolar fluid (K>0).

**Figure 6:**
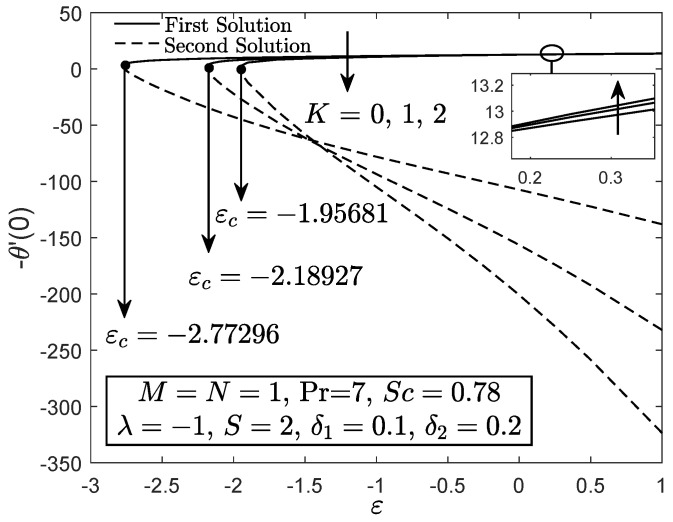
$-\theta^{\prime }\left(0\right)$ towards ε for viscous fluid (K=0) and micropolar fluid (K>0).

**Figure 7:**
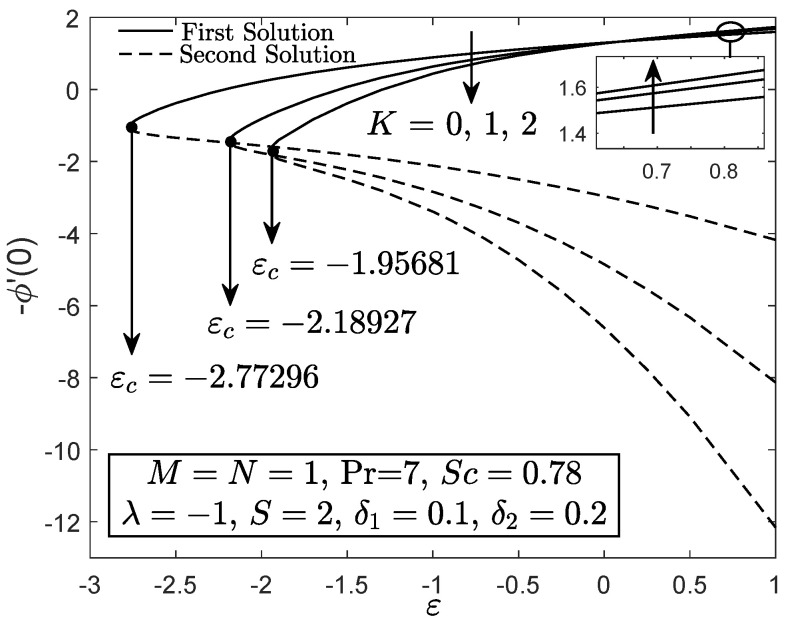
$-\phi^{\prime }\left(0\right)$ towards ε for viscous fluid (K=0) and micropolar fluid (K>0).

Figure 5 portrays the $f^{\prime \prime }\left(0\right)$ against ε for both Newtonian and micropolar fluids. The value of $f^{\prime \prime }\left(0\right)$ for the shrinking flow $\left(\varepsilon \to -\varepsilon \right)$ deteriorates as the material parameter *K* enhances while the contrary result is obtained as ε approximately greater than 0.14 (stretching flow). Figure 6 and Figure 7 elucidate that the micropolar fluid has greater heat and mass transfer rates for the stretching flow case as compared to the viscous fluid, whereas a contrast finding is obtained for the shrinking flow case. The results for stretching case are in good agreement with those by Rashad et al. [30]. The heat and mass transfer coefficients for micropolar fluid are greater as compared to the viscous fluid because the thermal and solutal boundary layer thicknesses decrease with the increment of micropolar parameter for the stretching case. In addition, it seems that the micropolar fluid has different behavior in stretching and shrinking cases. Thus, the micropolar fluid is profitable to control the fluid flow, temperature and concentration in the polymer processing [30]. It is clear from the results in Figure 5, Figure 6 and Figure 7 that the separation/critical point decreases with the enhancement of material parameter, which means that, in a dual stratified medium, a viscous fluid can hold the boundary layer separation as compared to the micropolar fluid.

**Figure 8:**
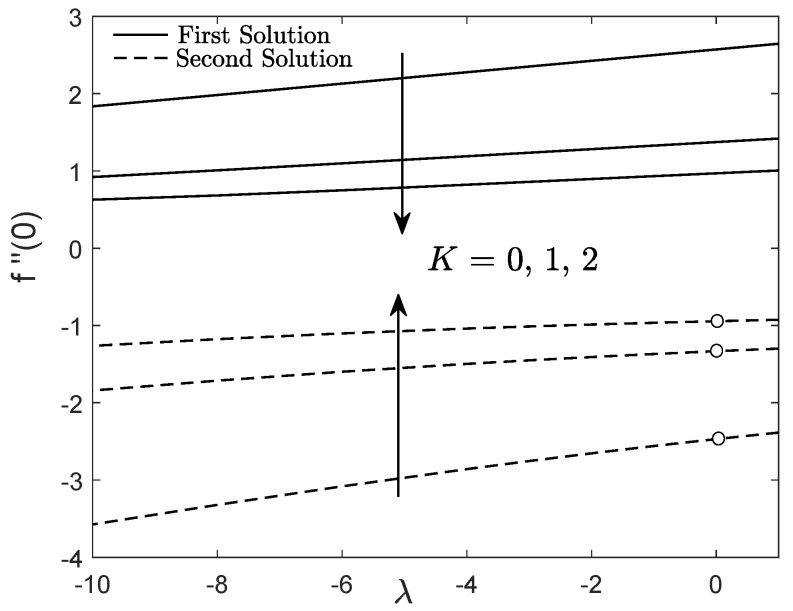
$f^{\prime \prime }\left(0\right)$ towards λ for viscous fluid (K=0) and micropolar fluid (K>0).

**Figure 9:**
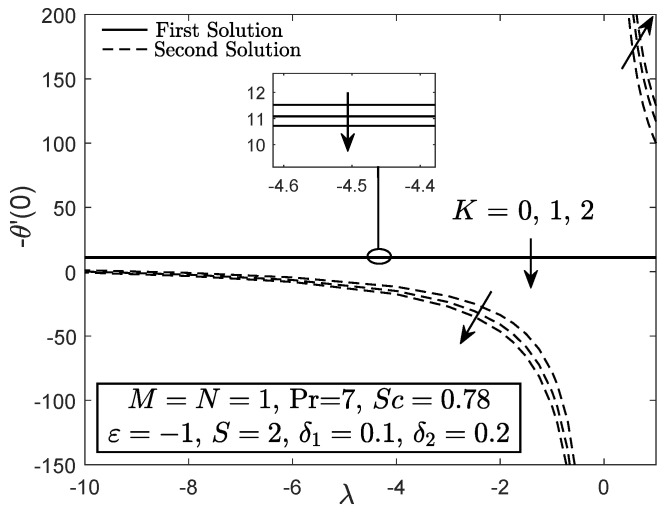
$-\theta^{\prime }\left(0\right)$ towards λ for viscous fluid (K=0) and micropolar fluid (K>0).

Most studies on mixed convective flow showed the presence of dual solutions for both assisting and opposing flow cases within a specific value of the buoyancy parameter, while a distinctive solution for pure forced convection flow. However, Figure 8 and Figure 9 only highlight the flow and heat transfer characteristics within the range of −10≤λ≤1. Higher magnitude of opposing buoyancy parameter (−10≤λ<0) and aiding buoyancy parameter (0<λ≤1) can induce two solutions, whereas the forced convective flow has a unique solution for each value of *K* (K=0,1,2). Both $f^{\prime \prime }\left(0\right)$ and $-\theta^{\prime }\left(0\right)$ of first solution diminish with the enhancement of the micropolar parameter for all type of convective flows (assisting, opposing and forced). This support the results in Figure 2, Figure 3, Figure 4, Figure 5, Figure 6 and Figure 7 that the micropolar fluid has low magnitude of skin friction coefficient, heat and mass transfer rates due to the application of the shrinking sheet.

**Figure 10:**
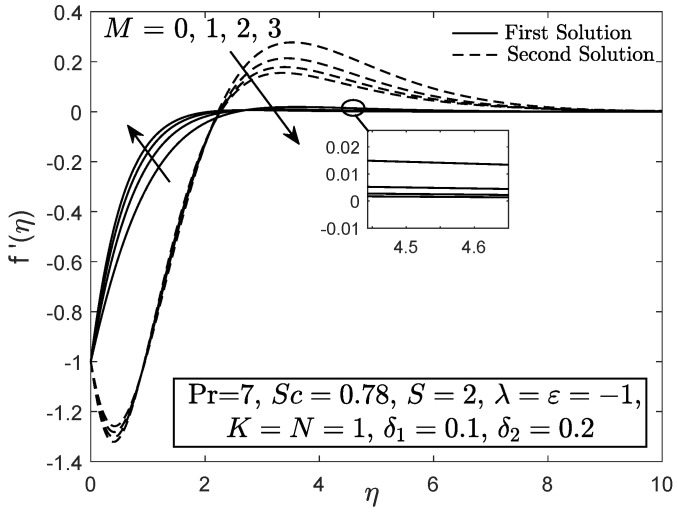
Velocity profile with magnetic parameter effect.

**Figure 11:**
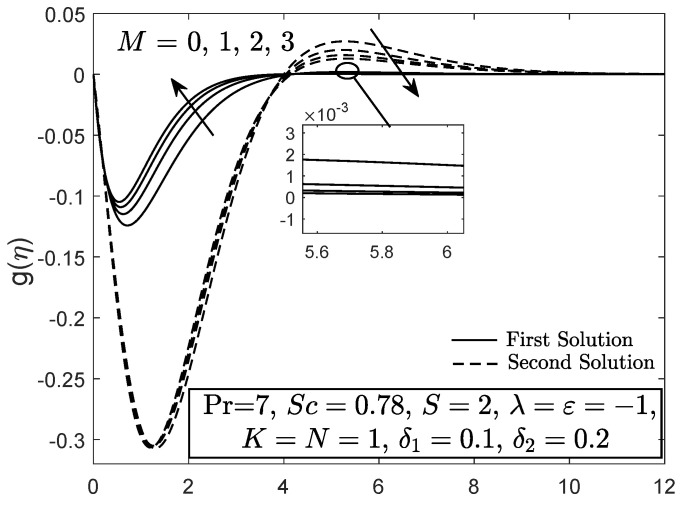
Angular velocity profile with magnetic parameter effect.

**Figure 12:**
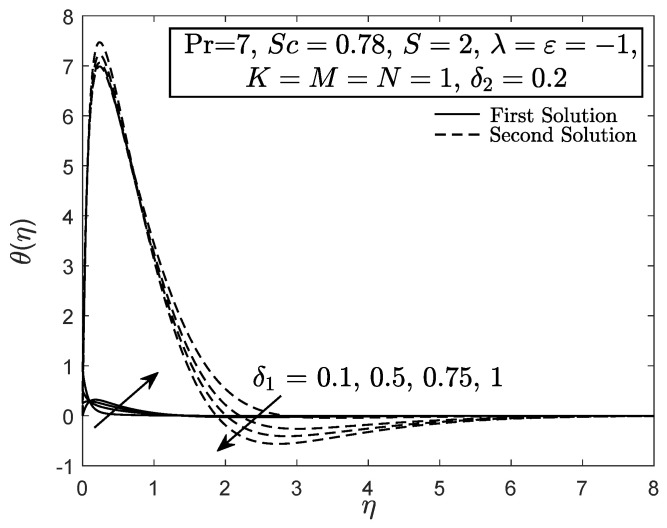
Temperature profile with thermal stratification effect.

**Figure 13:**
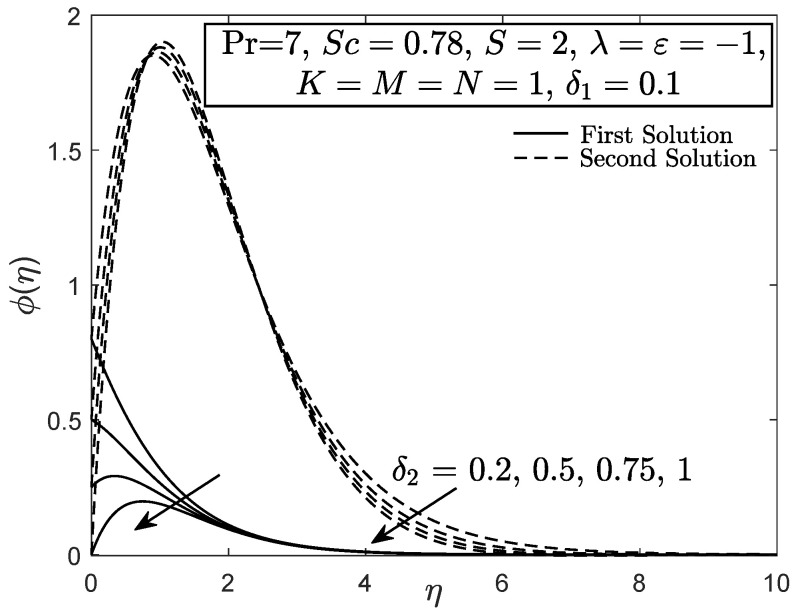
Concentration profile with solutal stratification effect.

**Figure 14:**
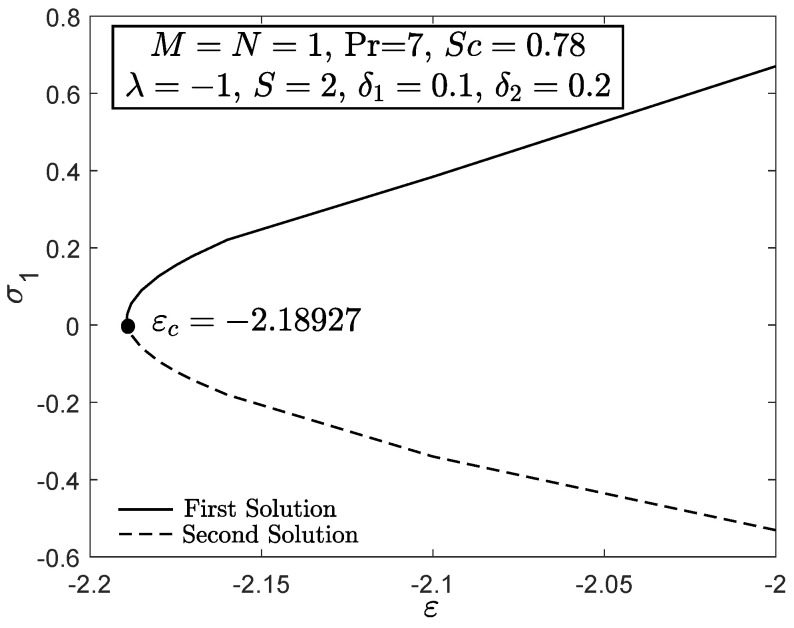
Smallest eigenvalue towards stretching/shrinking parameter.

Figure 10 and Figure 11 display the velocity and angular velocity profiles of the micropolar fluid with the imposition of magnetic parameter *M*. Both velocities slightly decrease as *M* is added when $\eta \to \eta_{\infty }$ while an initial growth of velocity is observed near the boundary. This phenomenon shows that the Lorentz force still gives small effect to the micropolar fluid. The fluid temperature for first solution enhances as the thermal stratification parameter $\delta_{1}$ is added, whereas the second solution has opposite characteristic, as exhibited in Figure 12. Both first and second solutions for the concentration profile diminish with the enhancement of the solutal stratification parameter $\delta_{2}$, as demonstrated in Figure 13. Figure 14 exemplifies the smallest eigenvalue $\sigma_{1}$ towards the stretching/shrinking parameter ε. It is clear that the first and second solutions have positive and negative eigenvalues, appropriately. It is also noticed that, as $\varepsilon \to \varepsilon_{c}$, $\sigma_{1}\to 0$, which validates the stability formulation in the present work and, subsequently, proves the reliability of the first solution.

## 5. Conclusions

The present work deals with the stretching/shrinking flow of a micropolar fluid with the mutual effects of suction, magnetic field and double stratification. The numerical computation was successfully performed using bvp4c function in Matlab software. Suction is one of the control parameters that is responsible for inducing the dual solutions for both flow cases. The implementation of stability analysis mathematically validates that the first solution is the real solution. The micropolar fluid has higher heat and mass transfer rate for the stretching flow case while a contradictory result is obtained for the shrinking flow case. The thermal stratification parameter enhances the fluid temperature, whereas the augmentation of solutal stratification parameter has reduced the concentration profile. The present work may generate ideas for other researchers (mathematicians/engineers) on selecting: (i) the right control parameters, which can optimize the heat and mass transfer rates for the industrial/technological demand; and (ii) the control parameters, which can possess non-unique solution so that the flow and heat transfer characteristics are not misinterpreted.

## Figures and Tables

**Table 1:** Comparison results for limiting cases between present model and Srinivasacharya and Upendar [28].

*M*	$-\theta^{\prime }\left(0\right)$		$-\phi^{\prime }\left(0\right)$		$\varepsilon_{a}=\left|\frac{a-b}{a}\right|\times 100\%$	
	Present	[28]	Present	[28]	$-\theta^{\prime }\left(0\right)$	$-\phi^{\prime }\left(0\right)$
0	0.62576	0.62289	0.28162	0.28042	0.4586%	0.4261%
1	0.55941	0.55703	0.23958	0.23895	0.4254%	0.2630%
2	0.51257	0.51043	0.21172	0.21176	0.4175%	0.0189%

$\varepsilon_{a}$ denotes the approximate percent relative error between present result, *a*, and previous result, *b*.

## Abbreviations

The following abbreviations are used in this manuscript:

$B_{0}$	magnitude of the magnetic field strength
$C_{0},T_{0}$	initial ambient concentration and temperature
$C_{w},T_{w}$	wall concentration and temperature
$C_{\infty },T_{\infty }$	ambient concentration and temperature
*K*	material parameter
*M*	magnetic parameter
*N*	solutal buoyancy parameter
Pr	Prandtl number
$Re_{x}$	local Reynolds number
*S*	suction parameter
*T*	fluid temperature
$u_{w}$	stretching/shrinking velocity
*t*	time	s
u,v	velocities along the *x*-, *y*-directions, respectively
α	thermal diffusivity of the fluid
θ	dimensionless temperature
λ	mixed convection parameter
μ	dynamic viscosity
ν	kinematic viscosity	$m^{2}/s$
ρ	density of base fluid	$\mathrm{kg}/m^{3}$
σ	unknown eigenvalue
$\sigma_{M}$	magnetic permeability of the fluid	$s^{3}A^{2}/\left(\mathrm{kg}m^{3}\right)$
τ	dimensionless time variable
$\tau_{1}$	ratio of the heat capacity of the nanoparticles to the base fluid
ϕ	dimensionless nanoparticle volume fraction/concentration
ω	microrotation or angular velocity
